# Advances in Diagnosis and Treatment in Otolaryngology

**DOI:** 10.3390/diagnostics15202606

**Published:** 2025-10-16

**Authors:** Lara Valentina Comini, Andrea Rampi, Stefano Bondi

**Affiliations:** 1 Head and Neck Oncology, Candiolo Cancer Institute, FPO-IRCCS, 10060 Candiolo, Italy; stefano.bondi@ircc.it; 2 Otorhinolaryngology Unit, Sondrio Hospital, ASST Valtellina e Alto Lario, 23100 Sondrio, Italy; andrea.rampi@asst-val.it

Over the last 20 years, otorhinolaryngology has undergone a gradual and considerable evolution. Technological innovations, novel imaging modalities, and emerging treatment strategies have significantly enhanced both the understanding and management of disorders affecting the head and neck region.

CT and MRI represent the gold standard for the diagnosis and follow-up of patients with chronic infectious diseases, as well as benign or malignant lesions of the head and neck. Their routine use in clinical practice, combined with more accurate image analysis and the specific training of radiologists primarily dedicated to ENT pathology, has led to improved diagnostic assessments and increasingly refined differential diagnoses of head and neck pathologies [1]. According to Mao K. et al. [2], comparison of the volume (ΔV%) of the two palatine tonsils may be clinically useful to help detect pathology on MRI in unknown primary head and neck tumors. In particular, patients with palatine cancer (PTC) showed a greater ΔV% than patients without PTC, with a cutoff value of 40%. Similarly, the accurate evaluation of postoperative CT and MRI images could prevent misdiagnosis in the case of pseudotumors induced by non-resorbed oxidized cellulose mimicking tumor recurrence [3].

Furthermore, a wide range of diagnostic tests have been proposed with the aim of guiding surgical dissection and enabling minimally invasive surgical interventions [4,5,6]. For instance, lymphoscintigrafy with 99mTc-nanocolloid and sentinel lymph node biopsy (SLNB), historically introduced into clinical practice for melanoma and breast cancer, currently represents a valid alternative to neck dissection in early oral squamous cell carcinoma, resulting in fewer functional complications for the patient, shorter operating and hospital stays, and lower costs [7]. Despite the encouraging results, some technical limitations inhibit the sensitivity of this method: in cases of cancer affecting the floor of the mouth or the base of the tongue, the presence of a level I lymph node may not be detected due to a phenomenon known as “shine-through radioactivity” [8]. To overcome this limitation, Galli et al. [9] recently proposed the combined use of 99mTc-Tilmanocept and indocyanine green fluorescence lymphangiography. In this initial two-case experience, 99mTc-Tilmanocept was found to be safe and effective for sentinel lymph node (SLN) identification in early oral squamous cell carcinoma OCSCC, where the high sensitivity provided by 99mTc-Tilmanocept was enhanced by the higher spatial resolution of ICG fluorescence.

In the era of minimally invasive and tailored surgery, the introduction of new technologies, combined with a growing interest in evaluating long-term surgical outcomes, is leading to individualized treatment plans aimed at achieving optimal results [10,11,12]. Robotic surgery is one of the most recent and promising advances in head and neck surgery. First proposed in 2005 by McLeod and Melder [13], the use of the da Vinci Robotic System was rapidly approved by the US Food and Drug Administration (FDA) for transoral procedures. Currently, the primary application of minimally invasive transoral robotic surgery (TORS) remains one of the main methods for the surgical removal of benign or malignant oropharyngeal and supraglottic lesions, in conjunction with surgery for obstructive sleep apnea syndrome (OSAS). Compared to the traditional transoral approach, robotic surgery can provide better exposure and visualization of unfavorable anatomical sites, allowing for more accurate and radical resection. A recent systematic review by Nocini et al. [12] analyzed the results of TORS for benign pathologies of the base of the tongue (BOT), highlighting its usefulness in terms of reducing recurrences in the treatment of congenital BOT masses, such as ectopic thyroid glands, dermoid cysts, and thyroglossal cysts. Similarly, TORS may have a disease-free survival advantage (*n* = 5 studies, RR: 1.13, 95% CI: 1.03, 1.24, I2 = 0%) compared to open surgery in oropharyngeal cancer, with no significant differences in mortality rate, recurrence rate, or positive margins [14]. Despite growing interest, data regarding the long-term oncological and functional outcomes of TORS are still scarce and scattered. A recent randomized clinical trial by Nichols et al. [15] prospectively compared post-treatment dysphagia and QoL in TORS versus radiotherapy (RT) in sixty-eight patients affected by early-stage oropharyngeal squamous cell carcinoma. Despite the inferiority of the robotic arm in terms of swallowing, pain, and dental problems at 1 year (patients on an oral diet in TORS + ND vs. RT at 1 year were 84 and 100%, respectively), long-term outcomes are almost comparable. Conversely, there is initial and conflicting evidence regarding the relationship between tumor location (tongue base, tonsil, or palate) and resection volume and functional outcomes of robotic surgery, as well as the impact of adjuvant therapies on swallowing performance [15,16,17,18].

While technological innovations in oncology are progressively improving the treatment of head and neck lesions, in the field of infectious diseases, international attention is focused on multidrug-resistant bacterial strains and their negative impact on patient outcomes and healthcare costs.

In 2023, the estimated total incidence of multidrug-resistant bloodstream infections was 10.35–3.97 per 100,000 inhabitants in Europe, and more than 35,000 people die each year in the Western world as a direct consequence of antimicrobial-resistant infections [19]. The prevalence of resistance is often higher in vulnerable patients, in whom antibacterial use is consequently more intense; for this reason, patients undergoing complex resections and free flap reconstructions for head and neck cancers should be considered at risk [20,21]. A recent study by Hamill et al. [22] analyzed the relationship between antibiotic prophylaxis and the prevalence of multidrug-resistant organisms in 147 patients undergoing head and neck cancer reconstruction. In total, 7 of the 30 postoperative infections (23.3%) were caused by multidrug-resistant bacteria, the most common of which was methicillin-resistant Staphylococcus aureus (MRSA), with no difference in the duration of antibiotic therapy.

Similar evidence comes from different geographic areas, with rates of drug-resistant postoperative infections ranging from 4 to 20%; however, further studies are needed to identify appropriate antibiotic prophylaxis and investigate predictors of infection in high-risk patients [22,23,24,25].

In parallel with this problem, climate change and migration flows are leading to the rediscovery of obsolete and underdiagnosed infections with head and neck manifestations, such as tuberculosis and zoonoses [26,27,28,29]. For instance, Hartl et al. [27] recently reported thirteen cases of tularemia in Tyrol. Even if rarely diagnosed in Central Europe, they detected antibodies against Francisella tularensis in 0.5% of healthy adults in Austria. The disease may be asymptomatic, but it can also give rise to various symptoms and require prolonged antibiotic therapy (mean of 5 ± 2 months) and hospitalization (median hospital stay of 13 days) in severe forms. For these reasons, in the case of unexplained head and neck symptoms, an evaluation for infectious diseases and screening for unusual infections is advisable, especially if empiric antibiotic treatment has been ineffective or if there is a specific medical history.

In conclusion, technological innovations are progressively improving the diagnosis and therapeutic options for head and neck diseases, with a growing focus on individualized and minimally invasive treatments. Specifically, robotic surgery is anticipated to significantly enhance future management strategies, with the principal aim to enhance radicality and reduce the impact of surgery. In the field of infectious diseases, on the other hand, we are observing an increase in postoperative complications linked to drug-resistant organisms and the rediscovery of obsolete and ignored pathologies.
